# Transverse prostate maximum sectional area can predict clinically significant prostate cancer in PI-RADS 3 lesions at multiparametric magnetic resonance imaging

**DOI:** 10.3389/fonc.2023.1082564

**Published:** 2023-02-20

**Authors:** Caterina Gaudiano, Lorenzo Braccischi, Makoto Taninokuchi Tomassoni, Alexandro Paccapelo, Lorenzo Bianchi, Beniamino Corcioni, Federica Ciccarese, Riccardo Schiavina, Matteo Droghetti, Francesca Giunchi, Michelangelo Fiorentino, Eugenio Brunocilla, Rita Golfieri

**Affiliations:** ^1^ Department of Radiology, IRCCS Azienda Ospedaliero-Universitaria di Bologna, Bologna, Italy; ^2^ Division of Urology, IRCCS Azienda Ospedaliero-Universitaria di Bologna, Bologna, Italy; ^3^ University of Bologna, Bologna, Italy; ^4^ Department of Pathology, IRCCS Azienda Ospedaliero-Universitaria di Bologna, Bologna, Italy; ^5^ Department of Specialty, Diagnostic and Experimental Medicine, University of Bologna, Bologna, Italy

**Keywords:** multiparametric magnetic resonance imaging, prostate cancer, PI-RADS 3 lesions, transverse prostate maximum sectional area, PIRADS 3, urological imaging

## Abstract

**Background:**

To evaluate multiparametric magnetic resonance imaging (mpMRI) parameters, such as TransPA (transverse prostate maximum sectional area), TransCGA (transverse central gland sectional area), TransPZA (transverse peripheral zone sectional area), and TransPAI (TransPZA/TransCGA ratio) in predicting prostate cancer (PCa) in prostate imaging reporting and data system (PI-RADS) 3 lesions.

**Methods:**

Sensitivity, specificity, positive predictive value (PPV) and negative predictive value (NPV), the area under the receiver operating characteristic curve (AUC), and the best cut-off, were calculated. Univariate and multivariate analyses were carried out to evaluate the capability to predict PCa.

**Results:**

Out of 120 PI-RADS 3 lesions, 54 (45.0%) were PCa with 34 (28.3%) csPCas. Median TransPA, TransCGA, TransPZA and TransPAI were 15.4cm^2^, 9.1cm^2^, 5.5cm^2^ and 0.57, respectively. At multivariate analysis, location in the transition zone (OR=7.92, 95% CI: 2.70-23.29, P<0.001) and TransPA (OR=0.83, 95% CI: 0.76-0.92, P<0.001) were independent predictors of PCa. The TransPA (OR=0.90, 95% CI: 0.082-0.99, P=0.022) was an independent predictor of csPCa. The best cut-off of TransPA for csPCa was 18 (Sensitivity 88.2%, Specificity 37.2%, PPV 35.7%, NPV 88.9%). The discrimination (AUC) of the multivariate model was 0.627 (95% CI: 0.519-0.734, P<0.031).

**Conclusions:**

In PI-RADS 3 lesions, the TransPA could be useful in selecting patients requiring biopsy.

## 1 Introduction

Proper management of patients with Prostate Imaging-Reporting and Data System (PI-RADS) 3 lesions on multiparametric Magnetic Resonance Imaging (mpMRI) represents one of the main unresolved problems in diagnosing prostate cancer (PCa).

The prevalence of PCa in PI-RADS category 3 lesions is highly variable, ranging from 7-15% (1, 2) to 25-46% (2) with a proportion of clinically significant PCa (csPCa) varying from 2 to 38%; this is the main factor limiting the choice between immediate biopsy and follow-up in this type of lesion.

In fact, the European Association of Urology (EAU) guidelines strongly suggest that patients having a PI-RADS score of 3 should undergo biopsy when the prostate-specific antigen (PSA) density is > 0.15 ng/ml/ml (3). The European Society of the Urogenital Radiology (ESUR) guidelines, together with PI-RADS version 2.1, suggest a more conservative approach for patients with a PI-RADS score of 3; these patients may undergo biopsy only if deemed appropriate according to clinical data and risk stratification (4). The aims of these approaches are to increase the detection rate of csPCas and, at the same time, to reduce the number of unnecessary biopsies and the exposure of patients with non-significant PCa to severe biopsy-related complications.

Recent studies (5–8) have demonstrated the effectiveness of using the mpMRI as a screening test before the first biopsy; however, some important issues should be defined, such as the correct selection of patients to undergo mpMRI or patients to undergo biopsy after performing an mpMRI. In particular, it is necessary to identify the most useful clinical data to make this selection and standardise their use in clinical practice.

Risk calculators have been introduced for this purpose; these are multivariable-based tools for the prediction of individual PCas and csPCa risk, based on population-based big data after histological verification which can suggest the need for biopsy. The risk calculators with the highest csPCa prediction are the European Randomized Study of Screening for Prostate Cancer (ERSPC) risk calculator, which was created from a European patient population, and the Prostate Cancer Prevention Trial (PCPT) risk calculator, which was created from a North American study, with the ERSPC risk calculator seemingly having greater PCa prediction capability (9, 10).

A recent study by Jiang S et al. (11) investigated new tools for predicting PCa in a cohort of patients who underwent mpMRI and biopsy, such as maximum sectional areas on transverse, coronal and sagittal T2-weighted (T2w) images of the entire prostate, and the peripheral and transition zones (PZ and TZ), separately. They found that prostate maximum sectional area (PA) is a good prostatic imaging parameter for predicting PCa, and the transverse sectional area of the central gland is significantly smaller in patients with PCa (p < 0.001) and has the greatest significant area under the receiver operating characteristic curve (AUC) (0.801) of all the predictors. The PA is manually segmented on the T2w scan and is related to prostate volume; however, it does not suffer from the limits of calculating the approximated prostate volume which is affected by the geometric distortion of the gland. Probably for this reason, the Authors demonstrated an outperformance of sectional areas compared to prostate volume and PSA density.

Starting from this experience, the aim of the present study was to evaluate the ability of new tools, such as TransPA (transverse prostate maximum sectional area), TransCGA (transverse central gland sectional area), TransPZA (transverse peripheral zone sectional area), and TransPAI (transverse prostate area index, i.e. TransPZA/TransCGA ratio) in predicting overall PCa and csPCa in a population of patients with PI-RADS 3 lesions at fusion-targeted biopsy (fusion-TB).

## 2 Materials and methods

### 2.1 Patients

This study was an observational, retrospective, single centre study; it was approved by our local Institutional Review Board (IRB), which waived the need for informed consent, and conducted in accordance with institutional guidelines, including the Declaration of Helsinki (Ethics Committee code: 784/2021/Oss/AOUBo).

One hundred forty patients with at least one PI-RADS 3 lesion, according to the ESUR guidelines version 2.1 at mpMRI performed at our Radiology Unit from September 2019 to December 2021 were enrolled.

The inclusion criteria were the following: 1) having undergone fusion-TB of the index lesion at our Radiology Unit and 2) having a histopathological report from a dedicated pathologist of the Pathology Unit of our institution.

The exclusion criteria were the following: 1) no concomitant PI-RADS 4 or 5 lesions; 2) mpMRI protocol not completely adhering to the suggested imaging protocols described in PI-RADS version 2.1; 3) active surveillance and previous surgical treatment, such as open simple prostatectomy (OP) or transurethral resection of the prostate (TURP); 4) the presence of severe artifacts, not allowing the evaluation of one or more sequences of the mpMRI due to uni/bilateral hip prostheses or other causes.

**Figure 1:**
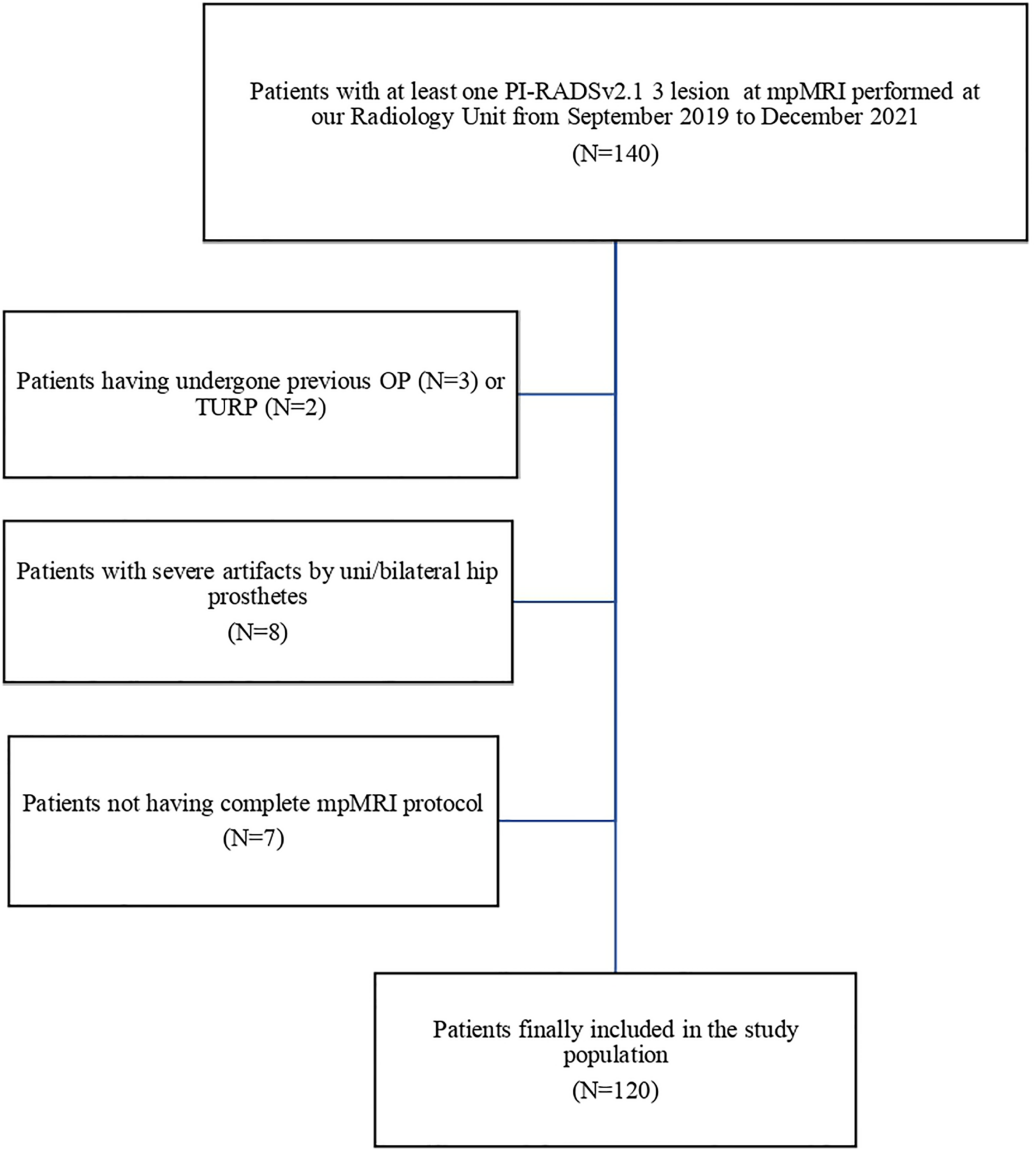
Flowchart for patient selection. PI-RADSv2.1, Prostate Imaging Reporting and Data System version 2.1; mpMRI, multiparametric Magnetic Resonance Imaging; OP, open simple prostatectomy; TURP, transurethral resection of the prostate.

After application of the exclusion criteria, 120 patients were included in the final analysis (**Figure 1**).

For each patient enrolled, clinical data, such as PSA, prostate volume, and PSA density, were collected.

### 2.2 Image acquisition and analysis

The mpMRI examinations were carried out using a 1.5T scanner (Signa HDxt; GE Healthcare, USA) and a pelvic phased-array surface coil combined with a disposable endorectal coil.

The multiparametric study of the prostate gland and seminal vesicles included Fast Relaxation Fast Spin Echo (FR-FSE) T2w, DWI (Diffusion Weighted Imaging) and Dynamic Contrast-Enhanced (DCE) sequences; the scan parameters have already been described in a previous study (9).

The DWI and DCE images were processed on an independent workstation with dedicated software (Functool, 4.5.5, GE Healthcare, USA) in order to obtain the ADC (Apparent Diffusion Coefficient) map and signal intensity-time (I-T) curve, respectively.

All the PI-RADS 3 lesions were catalogued according to location (peripheral or transition zone) and maximum diameter.

**Figure 2:**
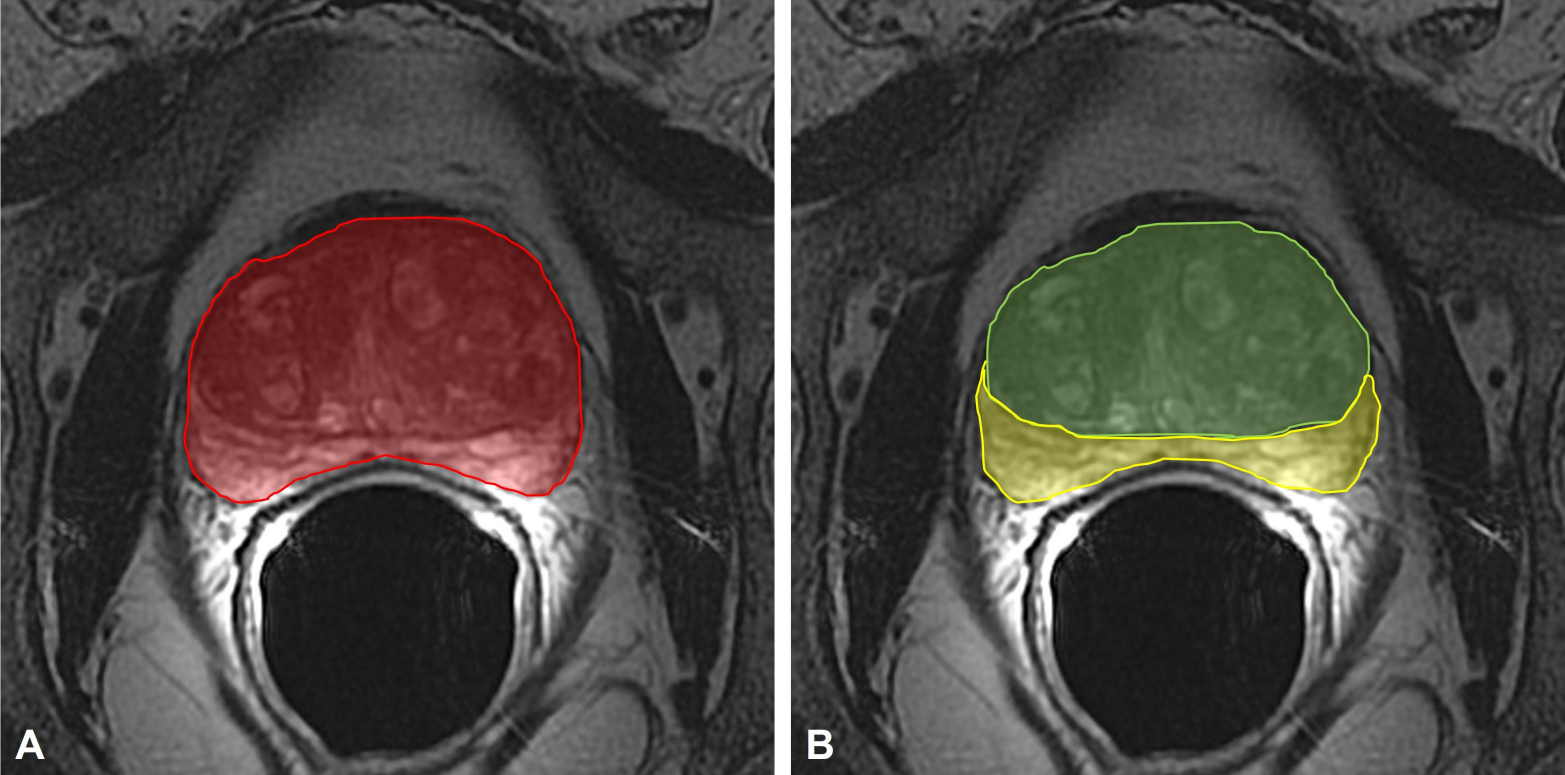
**(A)** measurement of the TransPA (red area) on the axial FR-FSE T2-weighted maximum sectional area of the prostate; **(B)** measurement of the TransCGA (green area) and TransPZA (yellow area) on the axial FR-FSE T2-weighted maximum sectional area of the prostate. TransPA, transverse prostate maximum sectional area; TransCGA, transverse central gland sectional area; TransPZA, transverse peripheral zone sectional area; FR-FSE, Fast Relaxation Fast Spin Echo.

Subsequently, the following parameters were measured on axial FR-FSE T2w sequences as previously described (11): 1) TransPA, 2) TransCGA, 3) TransPZA, and 4) TransPAI (**Figure 2**).

The prostate maximum sectional areas on the axial FSE T2w sequences were selected as follows; when the bilateral prostate lobes were basically symmetrical, and the quasi-circular internal urethral sphincter could be seen in the middle of the prostate, the maximum section was that in which the sectional area became smaller when scanning upward or downward.

The contouring of the total prostate area (TransPA) and the central gland area (TransCGA) were manually defined on this selected image; the peripheral zone area (TransPZA) was subsequently calculated.

### 2.3 Prostate biopsy and pathobiological analysis

All the PI-RADS 3 lesions were biopsied transrectally by two experienced radiologists, who usually read at least 400 mpMRI per year, using the mpMRI-TRUS Fusion image guide feature of “Aplio™ 500 ultrasound system”, after antibiotic prophylaxis and a cleansing rectal enema, using a non-disposable biopsy gun (Medgun, Medax, Modena, Italy) with a disposable 18-gauge needle and a US platform (Canon-Toshiba Aplio 500™, Ōtawara, Kanto, Japan) with an end-fire TRUS probe as previously described (10).

The biopsy samples were analysed by two dedicated genitourinary pathologists who primarily identified the presence or absence of a neoplastic pathology on the samples. Each neoplastic lesion was graded from 1 to 5 according to the International Society of Urological Pathology (ISUP) Grade Group System (GGS) (12); lesions having a GGS ≥ 2 were considered to be clinically significant.

### 2.4 Statistical analysis

Data were reported as medians, interquartile ranges (IQRs) and frequencies. A correlation between PI-RADS 3 lesions on mpMRI and fusion-TB results as in a per-lesion analysis was made. The diagnostic performance was evaluated using sensitivity, specificity, positive predictive value (PPV) and negative predictive value (NPV). The Mann–Whitney U test was used. The AUC was computed together with the 95% confidence interval (95% CI) and the asymptotic test for null hypothesis: true area=0.5. The best cut-off was calculated using maximisation of the Youden Index. Univariate and multivariate analyses were carried out using logistic regression. Odd ratios (ORs) were calculated together with their 95% CIs. The forward stepwise likelihood ratio method was used for variable selection in the multivariate analysis. The AUC was computed to evaluate the predictive accuracy of the multivariate model. All the tests were 2-tailed, and a p value of <0.05 was considered statistically significant. All the statistical analyses were carried out using IBM SPSS 25.0 (SPSS Inc., Armonk, NY, USA).

## 3 Results

**Table 1** summarises the demographic and clinical characteristics of the patient population and the pathological characteristics of the PCa lesions identified. The patient population had median age of 65.5 years, a median PSA of 6.7 ng/ml and a median prostate volume of 47.5 ml; 47 (39.2%) patients had a PSA Density ≥ 0.15 ng/ml/ml.

**Table 1:** Overall demographic and clinic characteristics of the patient population.

Patient number, n (%)	120 (100%)
Age (years)	
Mean (SD)	65.0 (7.0)
PSA (ng/ml)	
Mean (SD)	7.9 (5.9)
Prostate volume (ml)	
Mean (SD)	57.3 (32.1)
PSA density (ng/ml/ml), n (%)	
< 0.15 ≥ 0.15	73 (60.8%) 47 (39.2%)
Index lesion location, n (%)	
Peripheral Zone Transition Zone	90 (75.0%) 30 (25.0%)
Lesion maximum diameter (mm)	
Mean (SD)	11.5 (5.1)
Pathological findings, n (%)	
PCa Non-neoplastic lesions	54 (45.0%) 66 (55.0%)
ISUP classification, n (%)	
GGS 1 GGS 2 GGS 3 GGS 4 GGS 5	20 (16.7%) 21 (17.5%) 7 (5.8%) 4 (3.3%) 2 (1.7%)

SD, Standard deviation; PSA, prostate specific antigen; PCa, prostate cancer; ISUP, International Society of Urogenital Pathology; GGS, Grade Group System.

All the bold values are statistically significant (p value < 0.05).

One hundred twenty lesions in 120 patients were correctly identified on mpMRI, corresponding to the lesions identified on pathological specimens, and 90 (75.0%) were located in the PZ. Overall, 54 (45.0%) lesions were PCa while the remaining 66 (55.0%) were non-neoplastic lesions at fusion-TB; there were 34 (28.3%) csPCas (GGS ≥ 2).

The mpMRI parameters calculated on the prostate maximum sectional area on the axial FSE T2w sequences of the overall lesions were the following: the median TransPA was 15.4 cm^2^, the median TransCGA was 9.1 cm^2^, the median TransPZA was 5.5 cm^2^, and the median TransPAI was 0.57. **Table 2** shows the distribution of these parameters in the PCa and the non-neoplastic lesion groups; the median TransPA and the TransCGA of the PCa patients were significantly lower than those reported in the non-neoplastic patients (P<0.001 and P=0.001, respectively) while the TransPAI was significantly higher (P=0.022).

**Table 2:** Parameters measured on mpMRI.

Parameters	Non-neoplastic lesions (N=66)	PCa (N=54)	*p-value*
**TransPA (cm^2^)** **Median (IQR)**	17 (14-21.9)	13.5 (12-16.4)	**<0.001**
**TransCGA (cm^2^)** **Median (IQR)**	11.5 (8.2-16)	7.8 (6.5-11.1)	**0.001**
**TransPZA (cm^2^)** **Median (IQR)**	5.6 (4.5-6.8)	5.5 (4.3-6.1)	0.269
**TransPAI** **Median (IQR)**	0.5 (0.3-0.7)	0.7 (0.4-1)	**0.022**

TransPA, transverse prostate maximum sectional area; TransCGA, transverse central gland sectional area; TransPZA, transverse peripheral zone sectional area; TransPAI, TransPZA/TransCGA ratio; IQR, interquartile range.

All the bold values are statistically significant (p value < 0.05).

**Table 3** illustrates the univariate and multivariate analyses which predicted the presence of PCa in the total population of patients with PI-RADS 3 lesions at fusion-TB. The univariate analysis showed a significant correlation between PCa and the TZ location (P<0.001), prostate volume (P=0.002), PSA Density (P=0.004), TransPA (P<0.001), TransCGA (P=0.001), and TransPAI (P=0.022). At the multivariate analysis, only the TZ location (OR=7.92, 95% CI: 2.70-23.29, P<0.001) and TransPA (OR=0.83, 95% CI: 0.76-0.92, P<0.001) were independent predictors of PCa.

**Table 3:** Univariate and multivariate analyses to predict the presence of PCa at fusion-TB in the overall population of patients with PI-RADS 3 lesions.

	UNIVARIATE ANALYSIS		MULTIVARIATE ANALYSIS		
	OR (95% CI)	*p-value*	OR (95% CI)		*p-value*
**TZ location**	6.25 (2.42 – 16.19)	**<0.001**	7.92 (2.70 – 23.29)		**<0.001**
**PSA (ng/ml)**	1.00 (0.94-1.06)	0.887	–		–
**Prostate volume (ml)**	0.97 (0.95 – 0.99)	**0.002**	–		–
**PSA Density (ng/ml/ml)**	3.09 (1.45 – 6.62)	**0.004**	–		–
**Index lesion maximum diameter (mm)**	1.04 (0.97 – 1.12)	0.256	–		–
**TransPA**	0.85 (0.78 – 0.93)	**<0.001**	0.83 (0.76 – 0.92)		**<0.001**
**TransCGA**	0.86 (0.78 – 0.94)	**0.001**	–		–
**TransPZA**	0.89 (0.72 – 1.10)	0.269	–		–
**TransPAI**	3.44 (1.20 – 9.96)	**0.022**	–		–

PCa, prostate cancer; TB, targeted biopsy; PI-RADS, prostate imaging reporting and data system; TZ, transition zone; PSA, prostate specific antigen; TransPA, transverse prostate maximum sectional area; TransCGA, transverse central gland sectional area; TransPZA, transverse peripheral zone sectional area; TransPAI, TransPZA/TransCGA ratio; OR, odds ratio; CI, confidence interval.

All the bold values are statistically significant (p value < 0.05).

The symbol “-” means that the parameter is not statistically significant in the multivariate analysis according to the “forward stepwise likelihood ratio method”, which excludes each parameter until only the statistically significant ones remains.

Moreover, the univariate and multivariate analyses which predicted the presence of csPCa (GGS ≥ 2) in the overall population of patients with PIRADS 3 lesions at fusion-TB are reported in **Table 4**. Univariate analysis showed a significant correlation between csPCa and prostate volume (P=0.029), TransPA (P=0.022), and TransCGA (P=0.030). At multivariate analysis, only TransPA (OR=0.90, 95% CI: 0.082-0.99, P=0.022) was an independent predictor of csPCa.

**Table 4:** Univariate and multivariate analyses to predict the presence of csPCa (GGS ≥ 2) at fusion-TB in the overall population of patients with PI-RADS 3 lesions.

	UNIVARIATE ANALYSIS		MULTIVARIATE ANALYSIS	
	OR (95% CI)	*p-value*	OR (95% CI)	*p-value*
**TZ location**	2.06 (0.86 – 4.94)	0.105	–	–
**PSA (ng/ml)**	1.01 (0.94 – 1.08)	0.825	–	–
**Prostate volume (ml)**	0.98 (0.96 – 1.00)	**0.029**	–	–
**PSA Density (ng/ml/ml)**	2.21 (0.99 – 4.96)	0.054	–	–
**Lesion maximum diameter (mm)**	1.00 (0.92 – 1.08)	0.909	–	–
**TransPA**	0.90 (0.82 – 0.99)	**0.022**	0.90 (0.82 – 0.99)	**0.022**
**TransCGA**	0.90 (0.82 – 0.99)	**0.030**	–	–
**TransPZA**	0.94 (0.74 – 1.18)	0.565	–	–
**TransPAI**	1.97 (0.70 – 5.59)	0.201	–	–

csPCa, clinically significant prostate cancer; GGS, Grade Group System; TB, targeted biopsy; PI-RADS, prostate imaging reporting and data system; TZ, transition zone; PSA, prostate specific antigen; TransPA, transverse prostate maximum sectional area; TransCGA, transverse central gland sectional area; TransPZA, transverse peripheral zone sectional area; TransPAI, TransPZA/TransCGA ratio, i.e. transverse Prostate Area Index; OR, odds ratio; CI, confidence interval.

The symbol “-” means that the parameter is not statistically significant in the multivariate analysis according to the “forward stepwise likelihood ratio method”, which excludes each parameter until only the statistically significant ones remains.

The best cut-off of TransPA for csPCa was 18 (Sensitivity 88.2%, Specificity 37.2%, PPV 35.7%, NPV 88.9%). The discrimination (AUC) of the multivariate model was 0.627 (95% CI: 0.519-0.734, P<0.031).

## 4 Discussion

### 4.1 PI-RADS 3 lesions

Considerable variability in both the prevalence of PI-RADS 3 lesions and csPCa in the detection rate among studies has consistently affected the standardisation of the biopsy approach (i.e. when and how to proceed to biopsy).

In fact, PI-RADS 3 lesions comprise a wide range of benign (including acute or chronic prostatitis, post-atrophic hyperplasia and benign hyperplastic nodules) and malignant lesions, usually indistinguishable solely on the basis of mpMRI imaging features (13). However, in our series csPCas represent 28.3% of all PI-RADS 3 lesions; thus, a follow-up strategy could be considered safe and desirable as prostate biopsies are still affected by a non-negligible percentage of side effects, such as bleeding and infections (14).

### 4.2 Clinical parameters

Risk calculators, the most effective of which are the ERSPC and PCPT, can be utilised to predict the individual risk of csPCa before and after performing mpMRI in order to correctly select patients who require mpMRI in the first case and of biopsy in the second case. Artificial Intelligence (AI) can currently be applied to the use of risk calculations, allowing the processing of a large amount of data in a short time and providing predictive models useful in daily clinical practice, leading to the development of medication more and more tailored to the individual patient (15). Therefore, risk calculators can aid in selecting patients with PI-RADS 3 lesions at mpMRI who can benefit from follow-up or, alternatively, from immediate biopsy.

Of the clinical parameters, PSA density (with different cut-offs) represents the most investigated clinical predictor of PCa and a useful criterion in patient selection, especially in the grey area of PI-RADS 3 lesions (13, 16). To date, evidence has suggested that risk calculators and PSA density could be used in combination with risk stratification of patients with equivocal mpMRI results (15).

### 4.3 Radiological parameters

As regards the radiological features, in previous studies, lesion location (i.e. peripheral vs transition zone) has been correlated with an increased risk of PCa in which the peripheral location (17) or the transitional location (13) was an independent predictor of PCa. Other radiological features, such as T2w appearance, ADC value and/or DCE behaviour, do not allow a sufficient characterisation of benign vs. malignant lesions (13, 18, 19).

In the present study, at multivariate analyses, TransPA (OR=0.83, 95% CI: 0.76-0.92, P<0.001) was an independent predictor of all PCas together with lesion location (OR=7.92, 95% CI: 2.70-23.29, P<0.001). However, as regards only csPCas, the TransPA remains the only independent predictor (OR=0.90, 95% CI: 0.082-0.99, P=0.022) regardless of the lesion location, prostate volume, PSA and PSA density.

These results were in line with those reported by Jiang S et al. (11) who first proposed the introduction of these new tools. As suggested by the authors, the reason was possibly that the prostate is not a regular geometric solid and, therefore, any distortion, especially due to the growth mode of the tumor, could affect the calculation of the prostate volume. Moreover, prostate volume is usually estimated by an elliptical sphere formula (prostate volume=0.52 x length x width x height); any error regarding these measurements could be magnified by the multiplication (11).

The TransPA is a manual segmentation of the entire area of the prostate gland on axial T2w scans and is also very simple to define on any workstation for routine activity. However, with the advent of Computed Assisted Diagnosis (CAD) systems, it is much simpler to obtain this value as part of the standardised evaluation of the mpMRI exam and this parameter is integrated into the final report. Furthermore, a new generation of CAD has been implemented with the use of artificial intelligence (AI); it can support the diagnosis with the delineation of a probabilistic map of PCa for each lesion identified in the entire prostate gland (15). Artificial intelligence could be trained to obtain new tools, such as the TransPA, and integrate them into a predictive model of csPCa for each individual patient.

## 5 Limitations

The present study has some limitations. First, the results were obtained only at a single high-volume tertiary care centre, with significant experience in prostate mpMRI and fusion-TB. Therefore, generalising these results in a real-world setting requires caution. Second, the present study was subject to all the potential biases inherent in a retrospective study design, such as selection bias and limited sample size. Third, the definition of csPCa used in this study (GGS ≥ 2) may not be universally agreed upon as it did not account for tumour volume and was based on TB rather than on radical prostatectomy. Finally, our sample was heterogeneous and limited to 120 patients with PI-RADS 3 lesions; thus, multicentric studies with larger patient cohorts are needed to confirm the present results.

## 6 Conclusion

In conclusion, in the present study a new diagnostic parameter was identified, called TransPA, as a strong independent predictor of csPCa in a patient population of PI-RADS 3 lesions at fusion-TB. This parameter can be manually defined on the diagnostic workstation in a simpler manner as compared to prostate volume and PSA density and can be useful in selecting patients requiring prostate biopsy. However, additional studies are needed to confirm these data and integrate these results into clinical predictor risk models.

## Data availability statement

The raw data supporting the conclusions of this article will be made available by the authors, without undue reservation.

## Ethics statement

The studies involving human participants were reviewed and approved by Institutional Review Board of IRCCS Azienda Ospedaliero-Universitaria di Bologna (Ethics Committee 94 code: 784/2021/Oss/AOUBo, approved on 16 September 2021). The patients/participants provided their written informed consent to participate in this study. Written informed consent was obtained from the individual(s) for the publication of any potentially identifiable images or data included in this article.

## Author contributions

Conceptualization, CG, LBr, MT. Methodology, CG, LBr, MT, MD. Software, LBr, MT, AP. Validation, CG, LBr, MT, LBi, BC. Formal analysis, LBr, MT, MD, AP, FG. Investigation, CG, LBr, MT, LBi, BC. Resources, CG, LBr, MT, LBi, BC. Data curation, LBr, MT, AP, FG. Writing—original draft preparation, CG, LBr, MT, LBi, BC, FC, FG. Writing—review and editing, CG, LBr, MT, LBi, BC, FC, FG. Visualization, MF, RS, EB, RG. Supervision, MF, RS, EB, RG. Project administration, CG, RG. All authors contributed to the article and approved the submitted version.
